# Patient with ankylosing spondylitis and scleroderma renal crisis

**DOI:** 10.31138/mjr.29.2.86

**Published:** 2018-06-29

**Authors:** Aliki I. Venetsanopoulou, Ourania Argyropoulou, Athanasios G. Tzioufas

**Affiliations:** Department of Pathophysiology, Medical School, University of Athens, Athens, Greece

**Keywords:** Ankylosing spondylitis, Systemic Scleroderma

## Abstract

We report a 56-year-old man with a history of ankylosing spondylitis and systemic scleroderma. The patient had been diagnosed with ankylosing spondylitis 20 years ago and had been receiving treatment with NSAIDs and anti TNFα drugs. He referred to our rheumatology department for Raynaud’s phenomenon, arthralgias and weight loss. Physical examination revealed stiffness of the skin with difficulty in pinching (mainly at lower extremities, from knee to ankle). Soon after his first visit to our department, he developed renal scleroderma crisis with abrupt increase in blood pressure, decline in renal function, and microangiopathic haemolytic anaemia in accordance with positive antinuclear autoantibodies and positive anti-topoisomerase I antibody (anti-Scl70). This is one of the few reports in the literature of coexistence of ankylosing spondylitis and systemic scleroderma. A genetic correlation seems to be an explanation in some patients who carry one or two susceptibility alleles to both diseases. Thus, this might be the case of a ‘genetic trap’ in which distinct genes are cooperating to favour the susceptibility to two different HLA-associated systemic autoimmune diseases.

A 56-year-old man with a known history of ankylosing spondylitis (AS) was referred to our rheumatology department for Raynaud’s phenomenon, arthralgias and body weight loss (∼ 10% BMI). The patient had been receiving treatment for 10 years with an anti-TNFα agent. Six months before, due to his new symptoms, 200mg of hydroxychloroquine and 10mg of prednisolone per day were added. However, the patient showed no improvement and visited the external rheumatology department of the Clinic of Pathological Physiology of the Laikon General Hospital for a second opinion.

According to his medical history, he had ankylosing spondylitis that started with inflammatory back pain 20 years ago, with radiographic findings of sacroiliitis and spinal involvement (**Figures 1a, 1b**) and a positive HLA-B27 test. He had been receiving treatment with NSAIDs and anti TNFα drugs (adalimumab that was switched 6 months before to etanercept). He also suffered from depression and he had been treated with citalopram and alprazolam. He was a smoker (30 years, 1 pack a day), a social drinker and he had free family history.

**Figures 1a.**
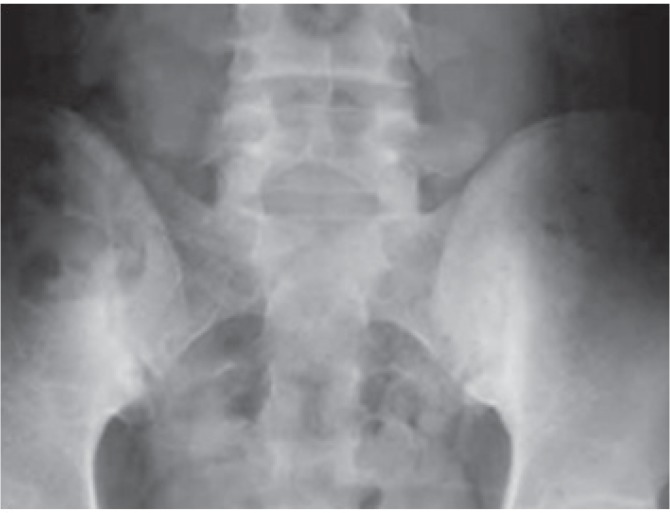
Sacroiliac joint radiography demonstrating bilateral sacroiliitis

**Figures 1b.**
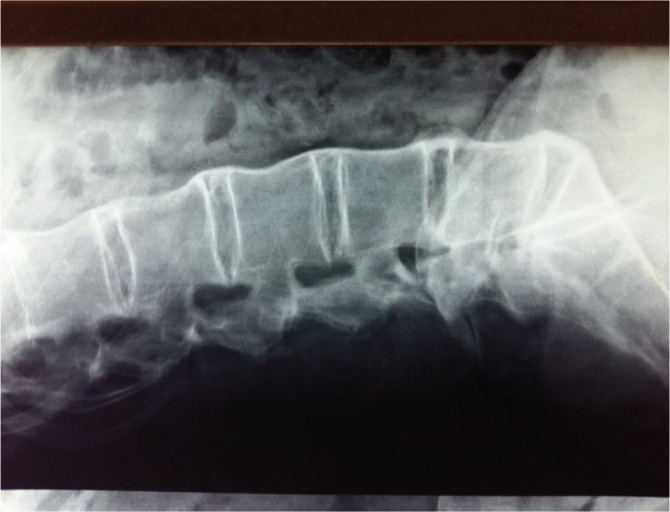
Lumbar spine X-ray with marginal syndesmophytes

On physical examination, Raynaud’s phenomenon was observed in both hands, with joint pain in all limbs and stiffness of the skin with difficulty in pinching (mainly at lower extremities, from knee to ankle). The rest of the physical examination and vital signs were normal. Laboratory tests were without pathological findings, with only an increase of ESR (57mmHg). Due to the significant weight loss and his smoking, a chest and upper/lower abdominal CT scan was requested. The results were without abnormal findings. Further evaluation for Raynaud’s phenomenon and stiffness of the skin included serum protein electrophoresis, hepatitis control and immunological tests (antinuclear autoantibodies [ANA], antibodies to Extractable Nuclear Antigens [ENA], rheumatoid factor, anti-topoisomerase I antibody [anti-Scl70] and cryoglobulins [CRYO]). While waiting for the results, hydroxychloroquine was discontinued, prednisolone was reduced to 7.5 mg per day and, as he suffered from arthralgias, 15mg of methotrexate and 5mg of folate per week were added.

After one month, on his scheduled appointment the patient presented with an exacerbation of his symptoms. In particular, he had arthralgias, dyspnoea and orthopnoea. Physical examination revealed an extension of skin hardening (trunk, upper and lower extremities) (**Figure 2a, 2b**), unexplained velcro-like inspiratory movements and increased blood pressure (200/100 mmHg). Laboratory tests were performed immediately, and rapid decline of renal function was revealed (Creat: 6.78 mg / dl, Urea: 75mg / dl), with microangiopathic haemolytic anaemia (Ht 29.3%, Hb 8.1 gr/dl, MCV 91) and increased inflammation markers (CRP 100 mg/dl, TKE 47 mmHg). The immunological tests were positive for ANA (1/1280 fsp), Anti-SSA/Ro and Anti-Scl-70. As he had rapidly deteriorating renal function, the patient was admitted to the clinic for further investigation and management.

**Figure 2a, 2b.**
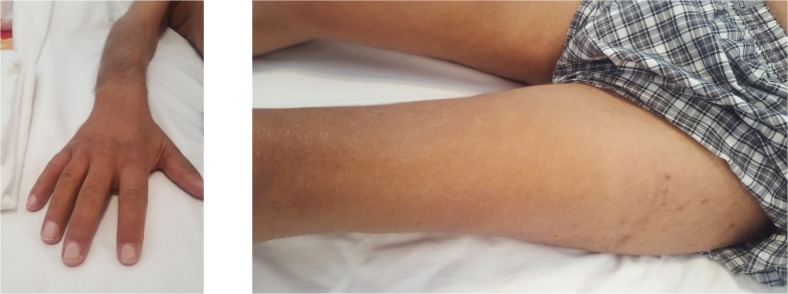
Stiffness of patient skin with difficulty in pinching

Based on his clinical picture and the abrupt increase in blood pressure, decline in renal function, microangiopathic haemolytic anaemia and, in accordance with the positive immunological tests, a diagnosis of scleroderma renal crisis was made. The patient was treated with an angiotensin converting enzyme inhibitor (captopril) in order to obtain a gradual adjustment of blood pressure. At the same time, he began haemodialysis sessions. However, he presented thrombocytopenia and leukopenia due to captopril and his treatment switched to an angiotensin II receptor blocker (valsartan). His blood pressure was successfully reduced to normal levels, but his creatinine remained high and he continued on haemodialysis. Currently, 1 year after his initial admission to the hospital, the patient remains on chronic haemodialysis and is treated for AS with a new biologic agent (secukinumab, a human interleukin-17A antagonist) at a dose of 150mg per month with satisfactory results and a BASDAI improvement.

## DISCUSSION

We report the case of a patient who had AS and developed diffuse systemic sclerosis (Ssc). Someone could suggest that scleroderma occurrence may be associated with our patient’s previous treatment. There are several factors have been identified as possible triggers of Ssc. These include drugs (e.g., vitamin K, cocaine, penicilla-mine and some chemotherapeutic agents), and chemicals (e.g., silica, pesticides, aliphatic hydrocarbons). However, the exact role of TNF-α drugs remains controversial. In vitro studies have shown that TNFα inhibits the activity of fibroblasts, acts as a potent inducer of metal-loproteinases and thus, inhibition of TNF-α could worsen fibrosis.^1,2,3^ On the other hand, inhibition of TNF-α seems to improve fibrosis in animal experimental models.^4^ Clinical studies have showed that, in some cases, anti-TNFα therapy worsened the fibrotic cellulitis and had infectious complications.^5^ Also, development of morphea, a localized scleroderma lesion, has been reported in patients treated with anti-TNFα drugs,^6,7,8^ suggesting a rare paradoxical side effect. However, in a small series of cases, anti-TNFα drugs are beneficial for the treatment of Ssc by improving arthritis and reducing the modified Rodnan score for skin (mRSS).^9^ Eventually, could this be a case of AS coexistence with Systemic Scleroderma? It is well known that AS rarely coexists with other inflammatory joint diseases. Cases of AS have been reported in patients who had systemic lupus erythematosus,^10^ mixed connective tissue disease,^11^ or Behçet’s disease.^12^ AS and Ssc rarely occur in combination.^13,14^ This is one of the few reports in the literature of coexistence of the two diseases. A genetic correlation seems to be an explanation. Particularly, there are reports of association of AS and SSc in the presence of HLA specificities known to be associated with these two diseases: HLA*B27, associated with susceptibility to AS; and HLA*B35, DRB1*11, DRB1*15, DRB3, DQB1*03, and DQB1*06, associated with SSc in different populations.^15^ Thus, this might be the case of a ‘genetic trap’ in which distinct genes are cooperating to favour the susceptibility to two different HLA-associated systemic autoimmune diseases. Hopefully, future research will identify the environmental factors that trigger the expression of both diseases in the same patient.
